# Bridging the Gap between Galectin-3 Expression and Hypertensive Pregnancy Disorders: A Narrative Review

**DOI:** 10.3390/jcm13164636

**Published:** 2024-08-08

**Authors:** Anastasios Potiris, Alexandros Fotiou, Eirini Drakaki, Angeliki Potetsianaki, Athanasios Zikopoulos, Efthalia Moustakli, Theodoros Karampitsakos, Spyridon Topis, Pavlos Machairoudias, Stamatoula Ouzouni, Angeliki Gerede, Panagiotis Christopoulos, Charikleia Skentou, Ekaterini Domali, Peter Drakakis, Sofoklis Stavros

**Affiliations:** 1 Third Department of Obstetrics and Gynecology, University General Hospital “ATTIKON”, Medical School, National and Kapodistrian University of Athens, 12462 Athens, Greece; alexandrosfotiou92@gmail.com (A.F.); thanzik92@gmail.com (A.Z.); theokarampitsakos@hotmail.com (T.K.); spyros.topis1996@gmail.com (S.T.); pavlosmach@gmail.com (P.M.); stama.ouz@gmail.com (S.O.); pdrakakis@med.uoa.gr (P.D.); sfstavrou@med.uoa.gr (S.S.); 2 First Department of Obstetrics and Gynecology, Alexandra Hospital, Medical School, National and Kapodistrian University of Athens, 11528 Athens, Greece; eirinidrak@med.uoa.gr (E.D.); kdomali@yahoo.fr (E.D.); 3 School of Education and Social Sciences, Frederick University, 1036 Nicosia, Cyprus; angelakp@yahoo.gr; 4 Laboratory of Medical Genetics, Medical School, University of Ioannina, University of Ioannina, 45110 Ioannina, Greece; thaleia.moustakli@gmail.com; 5 Department of Obstetrics and Gynecology, Democritus University of Thrace, 69100 Campus, Greece; agerede@otenet.gr; 6 Second Department of Obstetrics and Gynecology, Aretaieion University Hospital, Medical School, National and Kapodistrian University of Athens, 11528 Athens, Greece; panchrist@med.uoa.gr; 7 Department of Obstetrics and Gynecology, Medical School, University of Ioannina, 45110 Ioannina, Greece; haraskentou@uoi.gr

**Keywords:** gestational hypertension, preeclampsia, HELLP syndrome, Galectin-3 (Gal-3), placental insufficiency

## Abstract

Galectin-3 belongs to a family of soluble glycan-binding proteins, which are increasingly recognized as modulators of pregnancy-associated processes, including proper placental development. Gestational hypertension and preeclampsia are significant complications of pregnancy, affecting millions of women annually. Despite their prevalence, the underlying pathophysiological mechanisms remain poorly understood. Several theories have been proposed, including inflammation, placental insufficiency, disturbed placental invasion, and angiogenesis. The Scopus and PubMed/MEDLINE databases were utilized until the end of May 2024. In total, 11 articles with 1011 patients, with 558 in the control group and 453 in the preeclampsia group, were included. Seven articles investigated the expression of galectin-3 (Gal-3) in placental tissue samples, eight studies calculated the serum levels of Gal-3 in maternal blood samples, while one study referred to the possible correlation of galectin-3 levels in umbilical cord blood. The results were inconsistent in both the placental tissue and maternal serum; Gal-3 placental expression was found to be statistically increased in five studies compared to that in women without gestational hypertensive disorders, while two studies either mentioned decreased expression or no difference. Similarly, the Gal-3 maternal serum levels, compared to those in women without gestational hypertensive disorders, were found to be statistically increased in five studies, while three studies did not find any statistical difference. Gal-3 can play a crucial role in the pathogenesis of preeclampsia, and its expression is influenced by gestational age and placental insufficiency. A further investigation ought to be conducted to enlighten the correlation of Gal-3 with gestational hypertension and preeclampsia development.

## 1. Introduction

Gestational hypertension is a common complication of pregnancy, affecting approximately 5–10% of all pregnancies in the Western world [1]. Moreover, gestational hypertensive disorders are a significant cause of maternal and fetal mortality worldwide. The most severe complication of gestational hypertension disorders is the necessity for preterm delivery, which is associated with significant health risks, including stillbirth, neonatal mortality, delayed cognitive development in childhood, and an increased risk of metabolic and cardiovascular disorders later in life. The diagnosis of hypertension in pregnancy is based on measurements of blood pressure. Moreover, gestational hypertensive disorders can be divided into four different entities: pre-existing hypertension, gestational hypertension, preeclampsia, and eclampsia. Pre-existing hypertension is defined as hypertension preceding pregnancy or diagnosed before 20 weeks of gestation, while gestational hypertension usually develops after 20 weeks of gestation. Preeclampsia is actually gestational hypertension in combination with significant proteinuria (more than 300 mg per day) [1].

Despite the fact that gestational hypertension is such a common and simultaneously severe disorder of pregnancy and so much research has been conducted in order to enlighten its exact pathophysiology, the pathological pathway is still not clear. Several factors have been associated with gestational hypertensive disorders and preeclampsia [2]. The most common cause of gestational hypertensive disorders is the impaired remodeling of uterine spiral arteries or maternal or fetal vascular malperfusion. Other contributing factors include maternal conditions, such as systemic lupus erythematosus [3].

Galectins, a family of soluble glycan-binding proteins, are increasingly recognized as powerful modulators of pregnancy-associated processes, particularly in ensuring proper placental development [4,5,6]. At the moment, 15 galectins have been identified and have been investigated for their role in several pathological pathways. Among the galectin family members, galectin-1 (Gal-1), galectin-3 (Gal-3), and galectin-9 (Gal-9) are highly expressed at the fetal–maternal interface [7]. More specifically, several studies and published articles have demonstrated that gal-3 alterations were associated with impaired placental vascularization and perfusion, placental insufficiency, and therefore, preeclampsia development [8]. Moreover, galectins are believed to play crucial roles in reproductive processes, such as maternal–fetal immune tolerance, embryo implantation, and angiogenesis [8,9]. Galectin-3 plays a crucial role in the invasion and migration of trophoblast tissue, which are essential processes for successful implantation and placental development. A recent study demonstrated that galectin-3 is pivotal for the regulation of trophoblast cell behavior [10]. It was found that galectin-3 enhances the migratory and invasive capabilities of trophoblast cells by modulating various signaling pathways and cytoskeletal dynamics. The absence or downregulation of galectin-3 significantly impairs these processes, leading to inadequate placentation. This research underscores the importance of galectin-3 in maintaining normal trophoblast function and suggests that abnormalities in its expression or activity could contribute to gestational complications, such as preeclampsia and other hypertensive disorders. Evidence suggests that Gal-3 is one of the main participants in these processes, with other family members also contributing significantly to immune–endocrine interactions and maternal–fetal immunological responses.

Galectin-3 (Gal-3) is a multifunctional protein found in various tissues, including endothelial, epithelial, and immune cells, as well as sensory neurons [11,12]. It is overexpressed during the early stages of pregnancy and has been linked to adverse pregnancy outcomes [13]. Furthermore, the expression of galectin-3 is downregulated during life; during intrauterine embryonic life, there is an increased expression of galectin-3 that decreases as life progresses [14].

Galectin-3 is expressed by virtually all immunocompetent cells, including monocytes, macrophages, neutrophils, eosinophils, basophils, mast cells, and dendritic cells, and plays a crucial role in various immunological processes. Numerous studies have demonstrated the predominantly pro-inflammatory properties of galectin-3, which includes its role in recruiting macrophages and neutrophils, promoting phagocytosis, and enhancing the adhesion of granulocytes to endothelium [15].

Understanding the complex mechanisms underlying preeclampsia is crucial for developing effective diagnostic tools and interventions to prevent and manage this condition. Therefore, we conduct a narrative review of the literature to investigate and enlighten the possible correlation between galectin-3 and pregnancy pathologies such as gestational hypertension and preeclampsia.

## 2. Materials and Methods

The search strategy of this review adhered to the guidelines outlined in the Preferred Reporting Items for Systematic Reviews and Meta-Analyses (PRISMA) protocol [16]. Two authors (AP and AF) conducted the literature search and abstract selection independently. All clinical trials that investigated the possible correlation between galectin-3 and gestational hypertensive disorders, such as preeclampsia, eclampsia, or HELLP syndrome, were read. The present systematic review excluded case reports, reviews, animal studies, and publications in languages other than English. 

A comprehensive search was conducted across two major databases, PubMed/Medline (1966–2024) and Scopus (2004–2024), covering articles published up to March 2024 using the following search query: (preeclampsia OR eclampsia OR (hypertensive disorder*) OR HELLP) AND (Gal-3 OR galectin 3). Additionally, the reference lists of the retrieved full-text articles were searched to identify relevant studies in the field. Studies only published in English were included, while conference papers, editorials, and animal studies were excluded. Extracted data (sample studies, number of included patients, number of control patients, number of gestational hypertension patients, and results of each study) are displayed in Table 1.

## 3. Results

**Figure 1:**
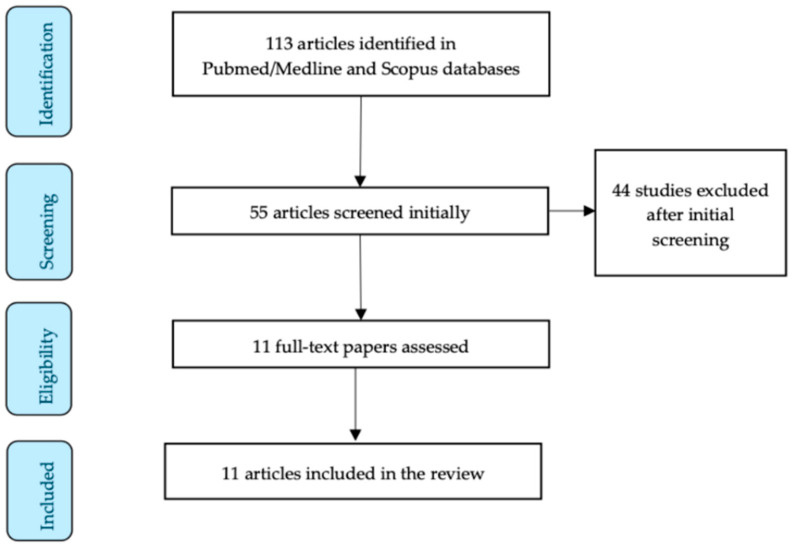
Study selection flow diagram.

The initial research resulted in 113 articles. The authors meticulously studied all of the abstracts independently, and finally, 11 articles were included in our review [7,17,18,19,20,21,22,23,24,25,26]. Figure 1 represents the study selection process of this narrative review.

Six studies were conducted in Europe, four studies in Asia, and one study in Australia. In our review, we included a total of 1011 pregnant patients. Of these, 558 patients were allocated to the control group, while 453 patients were assigned to the gestational hypertensive disorder/preeclampsia group. 

Table 1 shows the main characteristics and key outcomes of the included studies. Seven (7) studies investigated the expression of galectin-3 (Gal-3) in placental tissue samples, while eight (8) studies calculated the serum levels of Gal-3 in maternal blood samples. Moreover, one study investigated the levels of Gal-3 in umbilical cord samples. Regarding other patients’ characteristics, like gestational age, several articles did not refer to the exact ages of the included patients, and therefore, we did not include these details in our review. Despite this, in most cases, the control group patients’ gestational ages were matched to the gestational ages of the patients in the hypertension group.

The results of these studies were also inconsistent, with the expression of Gal-3 in the placental tissue samples being found to be statistically higher in five studies, decreased in one study, and not significantly different in one study. The maternal blood serum levels of Gal-3 were found to be significantly higher in five studies in the preeclampsia group compared to the control group, while three studies did not find statistically significant differences.

## 4. Discussion

This narrative review tries to enlighten the possible correlation of galectin-3 with gestational hypertensive disorders, such as preeclampsia. This review highlights the complexity of the relationship between galectin-3 and preeclampsia, with inconsistent results across studies.

Galectin-3 (Gal-3) is a multifunctional protein that plays a crucial role in various pathological processes. It is involved in pro-inflammatory signaling and interacts with various immune cells, including neutrophils, macrophages, and mast cells, thereby modulating immune functions [15]. Gal-3 has been shown to induce differentiation and angiogenesis in endothelial cells, which is closely linked to the pathogenesis of both preeclampsia and fetal growth restriction due to placental insufficiency [8]. Regarding fetal growth disturbances, several articles have linked the levels of galectin-3 expression with intrauterine growth restriction, a low birth weight, and the subsequent outcomes of these conditions, such as preterm labor and neurodevelopmental impairment. More specifically, Freitag et al. concluded in their study that the downregulation of galectin-3 was found to be significantly linked with abnormal placentation and fetal growth restriction afterwards [27]. This link is also based on several articles that correlate endometrial galectin-3 with embryo implantation [28].

As already mentioned, our review’s results are inconclusive. The included articles aimed to investigate the possible role of galectin-3 in gestational hypertensive disorders, such as preeclampsia. Most of the included studies found that placentas from women with preeclampsia exhibit an increased expression of Gal-3. However, one study demonstrated the downregulation of Gal-3 expression at the placental tissue level in early-onset preeclampsia [25]. This result could be explained due to the gestational ages of the included patients with preeclampsia. An early gestational age may be a potential confounding factor in the expression of Gal-3, as several studies have reported that Gal-3 levels are influenced by gestational age.

Regarding the serum levels of galectin-3, the results are inconsistent. In total, eight studies investigated these serum levels, with five studies reporting significantly higher levels in the patients in the preeclampsia group, while three studies did not find any significant difference. Interestingly, the only study that demonstrated a statistically significant downregulation of galectin-3 expression in the placental tissue of women with preeclampsia did not find a significant difference in the circulating levels of galectin-3 in women with early-onset preeclampsia compared to the control group [25]. Furthermore, all other included studies that evaluated both placental tissue galectin-3 expression and maternal serum levels mentioned upregulation and higher levels of galectin-3 in the serum in the gestational hypertensive disorder group. Table 1 demonstrates the results extracted from each included study.

The identification of galectin-3 as a biomarker for preeclampsia has important implications for the development of new diagnostic and prognostic tools for this condition. By accurately identifying women at risk of developing preeclampsia, clinicians may be able to implement early interventions and more closely monitor these patients, potentially reducing the risk of adverse maternal and fetal outcomes. Additionally, the use of galectin-3 as a biomarker may aid in the development of targeted therapies for preeclampsia, as it could help to identify specific pathways or mechanisms that contribute to the development of the disease.

Disturbed Gal-3 expression in preeclamptic placentas has been shown to impair placental function by affecting trophoblast biology. Gal-3 distortion may impair trophoblast invasion and vascularization, which could contribute to the development of preeclampsia [5]. Despite the pathogenetic factor of placental insufficiency, the investigated placentas in the included studies differed in their pattern of regulation of Gal-3 expression. The increased expression of galectin-3 in preeclamptic placentas that was found in most of the included studies of our narrative review (five studies out of seven) may be indicative of its involvement in the underlying mechanisms that contribute to the development of the disease. Galectin-3 has been shown to play a role in various cellular processes, including inflammation, angiogenesis, and trophoblast function, all of which are known to be dysregulated in preeclampsia. By exploring the specific mechanisms by which galectin-3 may contribute to the pathophysiology of preeclampsia, researchers may be able to identify new therapeutic targets and develop more effective interventions for this condition.

The potential mechanisms by which galectin-3 may contribute to the development of preeclampsia are multifaceted and involve its role in various cellular processes, including inflammation, angiogenesis, and trophoblast function [5,8,15]. Inflammation is a key feature of preeclampsia, and galectin-3 has been shown to play a pro-inflammatory role, promoting the recruitment and activation of immune cells, such as macrophages and neutrophils [11]. Additionally, galectin-3 has been linked to the regulation of angiogenesis, a process that is known to be dysregulated in preeclampsia [8]. The increased expression of galectin-3 in preeclamptic placentas may contribute to the impaired placental vascularization and perfusion that is characteristic of the disease.

Our narrative review was constructed upon a stringent review of the international literature, and it was intentionally designed to minimize potential article losses by eliminating date and language restrictions. Despite the well-structured research in the literature, our review has several limitations. The data were primarily sourced from few retrospective studies with small sample sizes, introducing the possibility of selection bias. Additionally, significant heterogeneity was noted among the included studies concerning the population, gestational age, and studied sample. Finally, the combined utilization of serum Gal-3 levels and Gal-3 expression in placenta samples to evaluate the risk for hypertensive disorders or preeclampsia was not feasible, thereby limiting the clinical applicability of our conclusions.

## 5. Conclusions

In conclusion, galectin-3 placental expression was found to be statistically increased in five studies, while two studies either mentioned decreased expression or no difference. Moreover, the maternal serum levels of Gal-3 were found to be statistically increased in five studies, while three studies did not find any statistical difference. Despite the fact that in both samples, the results were inconsistent, this review shows that galectin-3 can play a crucial role in the pathogenesis of preeclampsia, and its expression is influenced by gestational age and placental insufficiency. Further studies are needed to elucidate the mechanisms underlying the regulation of Gal-3 expression in these conditions and to identify potential therapeutic targets for the prevention and treatment of these disorders.

## Figures and Tables

**Table 1:** The main characteristics and key outcomes of the included studies.

Author (Year)	Sample	Total Patients’ Number	Control Group	PE Group	Main Outcomes
Jeschke et al. (2007) [7]	Placental tissue	27	8	19	Significant up-regulated staining in PE EVT cells
Lian et al. (2013) [17]	Placental tissue	154	105	49	No difference in placental expression between PE and control group; significant decreased expression of Gal-3 with increase in gestational age
Nikolov et al. (2020) [18]	Maternal blood samples	54	22	32	No significant differences in Gal-3 concentrations were observed between PE and control groups (*p* > 0.05)
Pankiewicz et al. (2020) [19]	Maternal blood and placental tissue samples	77	38	39	Serum galectin 3 levels were significantly higher in patients with PE compared to control group (*p* = 0.004); higher expression in placental tissue of PE group (*p* = 0.002)
Sattar Taha et al. (2020) [20]	Maternal blood samples	90	30	60	Gal-3 levels of PE group were significantly higher than that of control group (*p* = 0.017)
Gencheva et al. (2021) [21]	Maternal blood samples	123	50	73	Gal-3 levels were significantly higher in gestational hypertension and PE group compared to control group (*p* = 0.022 and 0.004, respectively)
Farladansky-Gershnabel et al. (2021) [22]	Maternal blood samples, umbilical cord blood samples, and placental tissue samples	30	20	10	In PE group, Gal-3 mRNA expression was significantly increased in maternal plasma, cord blood plasma, and placentas compared to control group (*p* < 0.05), and Gal-3 protein expression in placental tissue was statistically increased in PE group compared to control group (*p* < 0.05); maternal blood levels of Gal-3 were found to be statistically higher in PE group compared to control group (17.6 ± 1.4 vs. 13.6 ± 1.1 ng/mL, respectively; *p* = 0.03)
Ruikar et al. (2021) [23]	Placental tissue samples	60	30	30	Immunostaining of placental tissue confirmed increased expression of Gal-3 in PE group compared to control group; in Western blot, Gal-3 was increased by 3.14 fold (*p* = 0.031) compared to normal placenta, and mRNA expression of Gal-3 was increased in PE placenta compared to controls (*p* = 0.035)
Atakul et al. (2021) [24]	Maternal blood samples	80	35	45	No statistically significant difference between maternal serum levels between PE and control groups (979.5 ± 1394.3 vs. 1163.3 ± 1605.8 ng/mL, *p* = 0.122)
Kandel et al. (2022) [25]	Maternal blood and placental tissue samples	2 arms: 1 for preterm (64 patients) and 1 for term pregnancies (205 patients)	Preterm arm: 21, term arm: 182	Preterm arm: 43, term arm: 23	Gal-3 mRNA expression and protein were significantly decreased in placenta from pregnancies with early-onset PE compared to control group of preterm arm (*p* = 0.002 and 0.009, respectively); no significant difference in circulating Gal-3 levels in women with early-onset preeclampsia compared to controls (both preterm and term pregnancies)
Ghorbanpour et al. (2023) [26]	Maternal blood samples and placental tissue samples	47	17	30	Placental Gal-3 protein expression was increased in preeclampsia group (control 167.4 ± 56.7 vs. preeclampsia 498.2 ± 531.5, pg/mL, *p* = 0.004); moreover, plasma Gal-3 concentration from women with preeclampsia was also increased compared to controls (control 222.2 ± 72.91 vs. preeclampsia 288.8 ± 71.98, pg/mL, *p* = 0.004)
